# Cancer risk across mammals

**DOI:** 10.1038/s41586-021-04224-5

**Published:** 2021-12-22

**Authors:** Orsolya Vincze, Fernando Colchero, Jean-Francois Lemaître, Dalia A. Conde, Samuel Pavard, Margaux Bieuville, Araxi O. Urrutia, Beata Ujvari, Amy M. Boddy, Carlo C. Maley, Frédéric Thomas, Mathieu Giraudeau

**Affiliations:** 1 CREEC/CANECEV, MIVEGEC (CREES), University of Montpellier, CNRS, IRD, Montpellier, France; 2 Littoral, Environnement et Sociétés (LIENSs), UMR 7266 CNRS-La Rochelle Université, La Rochelle, France; 3 Institute of Aquatic Ecology, Centre for Ecological Research, Debrecen, Hungary; 4 Evolutionary Ecology Group, Hungarian Department of Biology and Ecology, Babeş-Bolyai University, Cluj-Napoca, Romania; 5 Department of Mathematics and Computer Science, University of Southern Denmark, Odense, Denmark; 6 Interdisciplinary Centre on Population Dynamics, University of Southern Denmark, Odense, Denmark; 7 Species360 Conservation Science Alliance, Bloomington, MN USA; 8 Laboratoire de Biométrie et Biologie Evolutive, Université de Lyon, Université Lyon 1; CNRS,UMR5558, Villeurbanne, France; 9 Department of Biology, University of Southern Denmark, Odense, Denmark; 10 Eco-Anthropologie (EA), Muséum National d’Histoire Naturelle, CNRS, Université de Paris, Musée de l’Homme, Paris, France; 11 Instituto de Ecologia, UNAM, Mexico City, Mexico; 12 Milner Centre for Evolution, Department of Biology and Biochemistry, University of Bath, Bath, UK; 13 Centre for Integrative Ecology, School of Life and Environmental Sciences, Deakin University, Geelong, Victoria Australia; 14 Department of Anthropology, University of California Santa Barbara, Santa Barbara, CA USA; 15 Arizona Cancer Evolution Center, Biodesign Institute and School of Life Sciences, Arizona State University, Tempe, AZ USA

**Keywords:** Cancer, Evolution, Zoology

## Abstract

Cancer is a ubiquitous disease of metazoans, predicted to disproportionately affect larger, long-lived organisms owing to their greater number of cell divisions, and thus increased probability of somatic mutations^1,2^. While elevated cancer risk with larger body size and/or longevity has been documented within species^3–5^, Peto’s paradox indicates the apparent lack of such an association among taxa^6^. Yet, unequivocal empirical evidence for Peto’s paradox is lacking, stemming from the difficulty of estimating cancer risk in non-model species. Here we build and analyse a database on cancer-related mortality using data on adult zoo mammals (110,148 individuals, 191 species) and map age-controlled cancer mortality to the mammalian tree of life. We demonstrate the universality and high frequency of oncogenic phenomena in mammals and reveal substantial differences in cancer mortality across major mammalian orders. We show that the phylogenetic distribution of cancer mortality is associated with diet, with carnivorous mammals (especially mammal-consuming ones) facing the highest cancer-related mortality. Moreover, we provide unequivocal evidence for the body size and longevity components of Peto’s paradox by showing that cancer mortality risk is largely independent of both body mass and adult life expectancy across species. These results highlight the key role of life-history evolution in shaping cancer resistance and provide major advancements in the quest for natural anticancer defences.

## Abstract (web summary)

An analysis of cancer mortality data for zoo mammals highlights marked differences across mammalian orders and an influence of diet, and shows that mortality risk is largely independent of body mass and life expectancy across species.

## Main

Complex multicellular organisms are built of millions to quadrillions of cells, ultimately all being derived from a single cell, the zygote. During the course of the organisms’ lifetime and owing to various mutational processes, cell lineages tend to accumulate mutations^7,8^. While the majority of mutations are harmless, some enable cells to escape cell cycle control, to grow and proliferate uncontrollably, resulting in cancer^9,10^. Cancer is a multistage process, where a set of mutations is required for both initiation and malignant progression. Given that every cell division carries a risk of generating mutations, organisms with large bodies (composed of more cells) and extended longevities (with a longer time to accumulate mutations) should be more likely to develop cancer^1,6,11,12^. Indeed, within humans^3,11^ and dogs^4^, larger individuals are more likely to develop cancer than smaller ones. Similarly, increasing age is one of the most potent carcinogenic factors in species in which cancer aetiology is well studied. Yet, while current evidence suggests that large body size and extended longevity result in increased cancer risk within species, this relationship may not hold across taxa^13^.

Limited data available so far indicate that vertebrates do not face clear size-dependent cancer risks despite their size and longevity varying by orders of magnitude. This poses a logical challenge, first formulated by Sir Richard Peto^6,14^. He noted that although mice have approximately 1,000 times fewer cells and >30 times shorter lifespans than humans, their risk of carcinogenesis is not markedly different (coined as Peto’s paradox)^5^. Peto’s paradox is an evolutionary conundrum that has puzzled the scientific community and has led to lively debate regarding the evolution of anticancer mechanisms. It is often postulated that natural selection on large size or extended longevity is inherently inseparable from the evolution of anticancer defences. Knowledge gained from investigating Peto’s paradox might thus largely contribute to our knowledge on natural anticancer mechanisms that could potentially be harnessed for medical use. Further, understanding cross-species variation of cancer vulnerability is an important next step in animal health and welfare. While a few studies aimed to establish cross-species variation in cancer risk^15,16^, most estimates and analyses have considerable limitations. These include small cross- or within-species sample sizes^1,6,17^, lacking information on the age distribution of cancer^15–17^, data heterogeneity (for example, biases due to domestication^17^ or combining data from multiple taxa^17,18^) or lack of control for phylogenetic relatedness among species^17^. Moreover, the effect of longevity was generally tested using the much-debated metric of maximum reported lifespan^15–17^. Nonetheless, cancer prevalence (the parameter exclusively considered by earlier studies^15–17^) is expected to correlate with life expectancy (the average time lived by individuals in the population of interest), not maximum lifespan potential (that veryfew individuals achieve), making these analyses inherently flawed.

To characterize cancer incidence in a homogeneous sample and across a wide taxonomic range, here we used the Zoological Information Management System (ZIMS), managed by Species360 (a non-profit organization custodian of zoo and aquarium data)^19^. We assembled information on 110,148 adult non-domesticated mammals distributed over 191 species, including data on their age, sex, dead/alive status and postmortem pathological records for 11,840 individuals. Cancer is registered in this database only for deceased animals and only if the inspecting veterinary pathologist considered it to be a factor that contributed to the individual’s death. First, to characterize species longevities, we used survival modelling (*n* = 110,148) and calculated species-specific adult life expectancies, representing average adult longevity in our sample^20,21^. Second, to estimate cancer mortality risk, we used two metrics, both estimating the proportion of individuals dying of cancer. We first calculated a simple measure of cancer mortality risk (hereafter CMR; that is, the ratio between the number of cancer-related deaths and the total number of individuals whose postmortem pathological records were entered in the database, *n* = 11,840), a measure adopted by earlier comparative studies^15,17^. This measure relies solely on dead individuals, ignoring the incomplete records of live animals, potentially introducing bias in cancer mortality estimates. Therefore, we also calculated the cumulative incidence of cancer mortality (hereafter ICM), a metric of cancer mortality risk eliminating potential biases due to disregarding left-truncation (that is, cancer before individuals enter the study) and right-censoring (individuals alive, thus with unknown fate at data extraction). Using these two metrics, we explored the phylogenetic distribution of cancer-related mortality across mammals. We then investigated Peto’s paradox and tested whether cancer mortality risk is associated with body size or the mean number of years lived by adults in the explored populations (that is, adult life expectancy).

## Cancer across the mammalian phylogeny

**Fig. 1:**
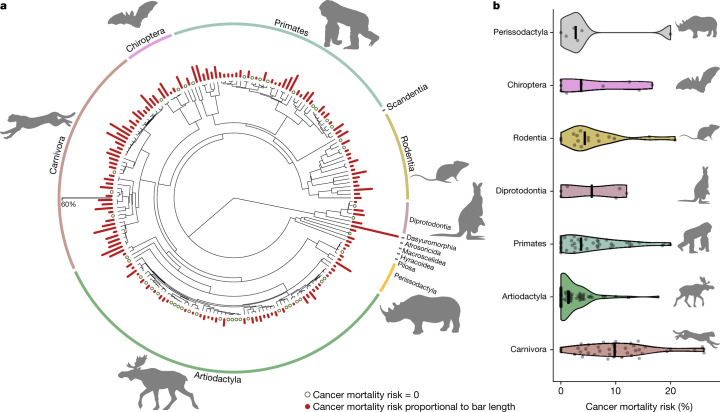
Distribution of cancer mortality risk across the mammalian phylogeny. **a**, CMR in various mammals (scale to bar plots is provided on the left of the graph). **b**, Violin plots indicating order differences in CMR in orders with a minimum of two species represented. Solid black lines represent order medians. Animal silhouettes used to visually represent mammalian orders were downloaded from PhyloPic (http://www.phylopic.org).

CMR was highly variable among species, ranging from 0% (in 47 species out of 191) to 57.14% in the kowari (*Dasyuroides byrnei*). CMR exceeded 10% in 41 species (21.5% of all species inspected), indicating that the oncogenic process is a prevailing source of mortality of many mammalian species distributed along the phylogeny, at least in managed populations (Fig. 1). ICM showed strong consistency with CMR (Pearson correlation test, *r* = 0.89, *t* = 25.14, df = 170, *P* < 0.0001). Nonetheless, all models were performed using both metrics to test for consistency in the results.

**Extended Data Fig. 1:**
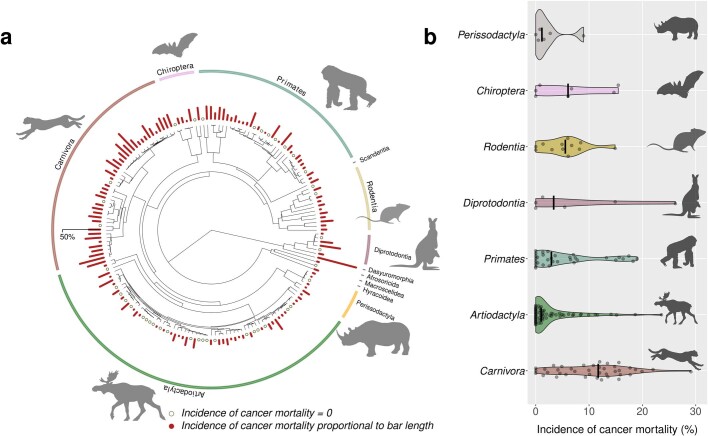
Phylogenetic distribution and order differences of ICM. **a**, Phylogenetic distribution of ICM (%). **b**, Violin plots indicating order differences in ICM (%) in orders with minimum two species assessed. Solid black lines indicate order medians. Animal silhouettes used to visually represent mammalian orders were downloaded from PhyloPic (http://www.phylopic.org).

Cross-species variation in cancer risk showed strong phylogenetic signal^22^ (CMR: *n* = 191, *λ* = 0.87, *P* < 0.0001; ICM: *n* = 172, *λ* = 0.69, *P* < 0.0001). To explore this, we compared cancer risk among mammalian orders represented by at least two species using linear regressions. Results indicated that the phylogenetic signal was mostly driven by cancer mortality risk in Carnivora, which was significantly higher than in Primates or in Artiodactyla (Extended Data Table 1, Fig. 1 and Extended Data Fig. 1). Both cancer mortality risk metrics indicated that Artiodactyla is the least cancer-prone mammalian order, despite the frequency of large-bodied species in this group (Extended Data Table 1).

High cancer risk in managed populations of Carnivora has previously been reported^23,24^. Possible explanations include the use of hormonal contraception (for example, progestins) and pregnancy postponement in zoo carnivores, both being significant risk factors for certain cancers in humans as well as non-domestic felids^24–26^. Nonetheless, if contraception was the key factor driving elevated cancer risk in Carnivora, a significant sex bias in cancer risk would be expected in this group, because hormonal contraception is usually administered to females. To test for sex bias in cancer risk across Carnivora, we estimated sex-specific CMR and ICM (only species with a minimum of ten males and ten females with available postmortem pathological records: *n* = 36 and *n* = 30 species, respectively). Pairwise comparison between sexes revealed no sex bias in either measure of cancer mortality risk (phylogenetic paired *t*-tests, CMR: *t* = 0.52, df = 33, *P* = 0.6061; ICM: *t* = −0.6815, df = 27, *P* = 0.5014) (ref. ^23^). Therefore, the generally high cancer risk in Carnivora is unlikely to be driven solely by the carcinogenic effects of reproductive management in zoo populations.

A high-fat, low-fibre diet, a known risk factor for carcinogenesis, has also been suggested to explain the elevated cancer risk in Carnivora^26,27^. Moreover, carnivores are on the top of the food chain, exposing them to bio-magnified effects of carcinogenic compounds^28^, such as pollutants^26^. Importantly, the consumption of raw meat can also expose carnivores to pathogens that can drive oncogenic transformation^29^. For instance, in humans it was estimated that 10–20% of all cancers are of viral origin^30^. While this figure is unknown in any other animal species^29^, it is arguable that raw meat consumption might exacerbate the spread of carcinogenic pathogens^31^. Exploring the association between diet and cancer risk could help to disentangle the influences of these risk factors.

**Fig. 2:**
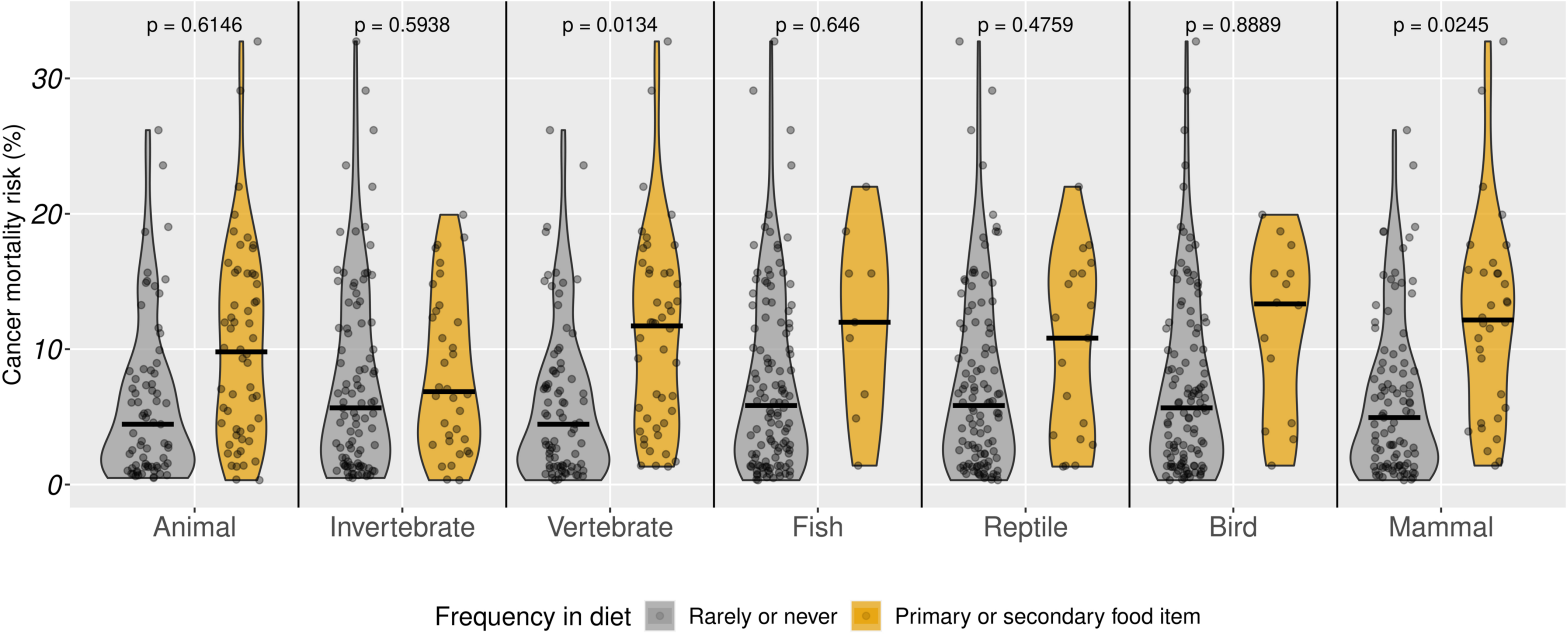
Cancer mortality risk in mammals as a function of animal content in diet. Violin plots show CMR as a function of seven diet items, each coded as rarely/never occurring in the diet or representing the primary/secondary food item of the species. Medians are marked with solid black lines. *P* values indicate pairwise differences as indicated by models presented in Extended Data Table 2 that also control for body mass, life expectancy and phylogeny.

**Extended Data Fig. 2:**
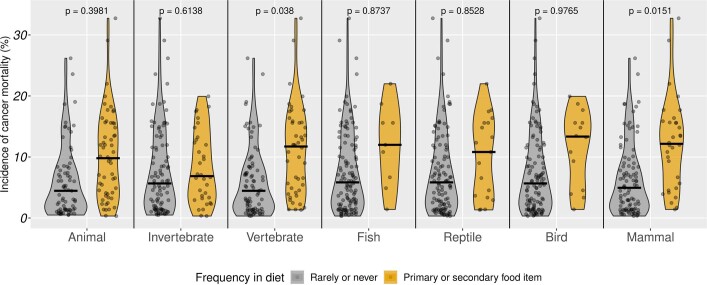
ICM in function of diet animal content. Violin plots indicate ICM in function of diet animal content. Diet is characterized by variables on three taxonomic levels: I. animal content (including any vertebrate or invertebrate prey); II. invertebrate or vertebrate prey; and III. within vertebrates fish, reptile, bird and mammal preys. Each variable is coded as a two-level factor: rarely/never occurring in diet and representing the primary/secondary food item of the species. Plots indicate the range and distribution of cancer risks in each category, where the width of each curve corresponds with the approximate frequency of data points in each region. Medians are marked with solid black lines. P values indicate pairwise differences as indicated by models presented in Extended Data Table 2 that also control for body mass, life expectancy and phylogeny.

**Extended Data Fig. 3:**
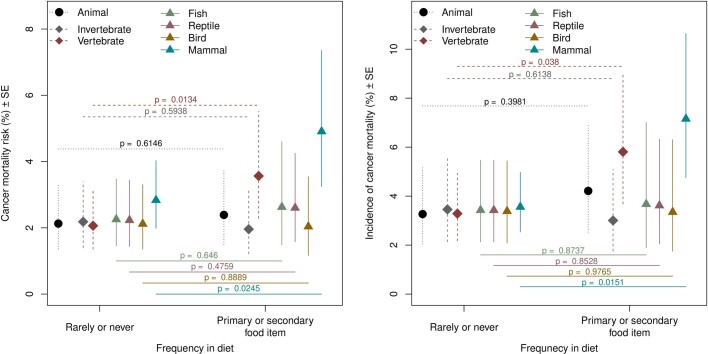
Predicted cancer mortality risk in function of animal content in diet. Values represent estimated marginal means of **a**, CMR or **b**, ICM in function of diet animal content, based on models presented in Extended Data Table 2. Diet is characterized by variables on three taxonomic levels: I. animal content (including any vertebrate or invertebrate prey); II. invertebrate or vertebrate prey; and III. within vertebrates fish, reptile, bird and mammal preys. Each variable is coded as a two-level factor: rarely/never occurring in diet and representing the primary/secondary food item of the species. P-values shown were obtained from models presented in Extended Data Table 2.

To explore the link between carnivorous diet and cancer risk, we collected data on the species’ natural diet (that is, consumption of animals, including invertebrates or vertebrates, and specifically of fish, reptiles, birds and mammals) from the literature^32^. Phylogenetic generalized least-squares (PGLS) regressions controlling for differences in longevity and body mass run separately for each diet item (Fig. 2, Extended Data Figs. 2 and 3 and Extended Data Table 2) indicated that species with animal-based diets have comparable cancer mortality risks (both CMR and ICM) to species that rarely or never consume animals. Nonetheless, consumption of vertebrate but not invertebrate prey was associated with increased cancer mortality risk. Specifically, mammals frequently consuming mammalian prey had significantly higher cancer mortality risk compared to mammals that rarely or never consume other mammals. Similar differences could not be detected in the case of fish, reptile or bird prey frequencies. These results indicate that a carnivorous diet has significant costs in terms of heightened oncogenic predisposition across mammals, particularly for diets high in mammalian prey. The nonsignificant association between cancer mortality risk and diet content of invertebrate, fish, reptile or bird indicates bio-magnification as a less likely source of elevated cancer risk among Carnivora. Nonetheless, the limited number of species primarily consuming these preys in our sample does not allow for definitive conclusions regarding this hypothesis. By contrast, the result that mammals consuming other mammals appear to have the highest cancer risk of all diet categories is consistent with a pathogenic origin of elevated cancer mortality risk among Carnivora. Host jumping of pathogens is most likely to occur in the case of phylogenetic proximity between the reservoir prey and the predator species^33^, making a mammal-to-mammal transmission the most likely host jump scenario. These results suggest that pathogen-driven oncogenesis might have a considerable role in shaping cancer mortality risk in mammals and urges the search for pathogens in various cancer types, while considering the notorious difficulty of proving oncogenic properties of pathogens^30^. Alternatively, high cancer risk in carnivorous animals might be related to their low microbiome diversity^34^, limited physical exercise under human care or other aspects of their physiology. Nonetheless, a lack of bias in these results, caused by potential alterations in the diet of housed carnivores, should be confirmed by studying natural populations. Importantly, these results probably reflect a complex, maybe indirect evolutionary link between diet and cancer vulnerability; therefore, the effect of meat consumption on cancer risk should be interpreted with caution.

## Test of Peto’s paradox

**Extended Data Fig. 4:**
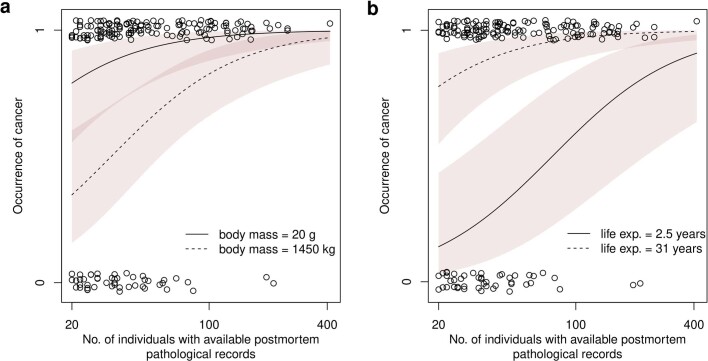
Association between occurrence of non-zero CMR and a, body mass or b, adult life expectancy across species. Occurrence of cancer in each species is plotted in function of the number of deceased individuals for which cause of death was known. Predictions were obtained for two scenarios, one for low and one for high **a**, body masses and **b**, adult life expectancies respectively. Random noise was added to cancer occurrence to facilitate the visualization of overlapping points. Predictions and associated 95% confidence intervals were obtained from a binomial GLM without phylogenetic control.

**Extended Data Fig. 5:**
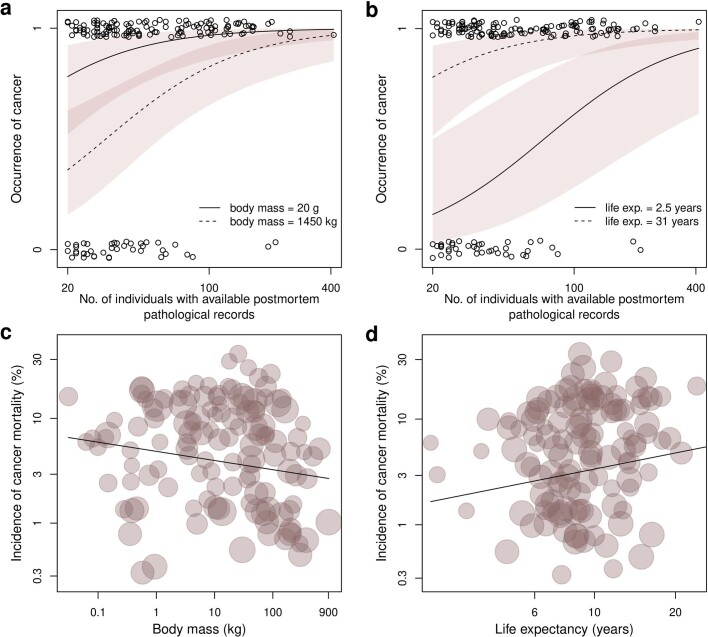
Association between ICM and a,c, body mass or b,d, adult life expectancy. Occurrence of cancer is plotted in function of the number of deceased individuals for which cause of death was known and predictions were obtained for two scenarios, one for low and high **a**, body masses and **b**, adult life expectancies respectively. Random noise was added to cancer occurrence to facilitate the visualization of overlapping points. Predictions and associated 95% confidence intervals were obtained from a binomial GLM without phylogenetic control. Non-zero cancer mortality risks were plotted against **c**, body mass or **d**, adult life expectancies. Slopes were obtained from the PGLS model presented in Extended Data Table 3b. Points are proportional to the log number of individuals with known cause of death.

Owing to the large number of zero cancer mortality risk estimates and thus non-Gaussian distributions, cancer risks were analysed using zero-inflated phylogenetic models (Methods), as a function of sample size, body mass and life expectancy. The probability of detecting at least one individual with cancer in a species increased steeply with increasing number of individuals with available postmortem pathological records (Extended Data Table 3). In fact, cancer was detected in at least one individual in almost all species with more than 82 individual pathological records available (Extended Data Figs. 4 and 5). Exceptions were the blackbuck (*Antilope cervicapra*) and the Patagonian mara (*Dolichotis patagonum*), where no cancer was detected despite postmortem pathological records being available for 196 and 213 individuals, respectively. This highlights the quasi-universality of oncogenic phenomena across mammals, illustrating that with adequate sampling, cancer is likely to be detected in all mammals. Our results further emphasize the fact that some members of the order Artiodactyla, besides rodents, are particularly cancer-resistant. Rodents have long been subject to scrutiny in the search for natural cancer resistance mechanisms^35^, owing to notoriously low cancer incidence in some species^36^. Nonetheless, cancer mortality risk in our dataset was lowest among ruminants, complying with rare cancer case reporting in this taxonomic group^15,37^. This indicates that other mammalian groups, especially Artiodactyla, might serve as informative model organisms in cancer research.

**Fig. 3:**
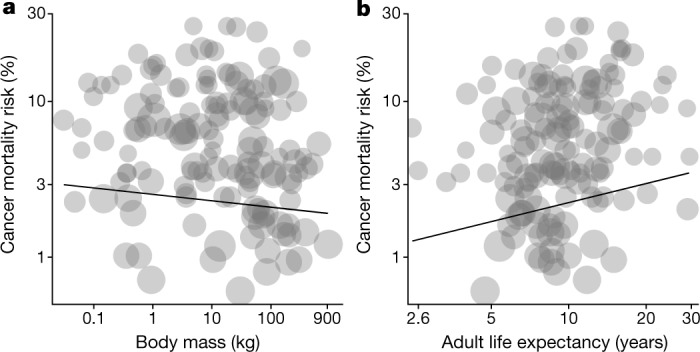
Association between cancer mortality risk and body mass or adult life expectancy across mammals. **a**, **b**, Non-zero CMR plotted against body mass (**a**) or adult life expectancies (**b**). Slopes were obtained from the PGLS model presented in Extended Data Table 3a. Points are proportional to the log number of individuals with available postmortem pathological records.

**Extended Data Fig. 6:**
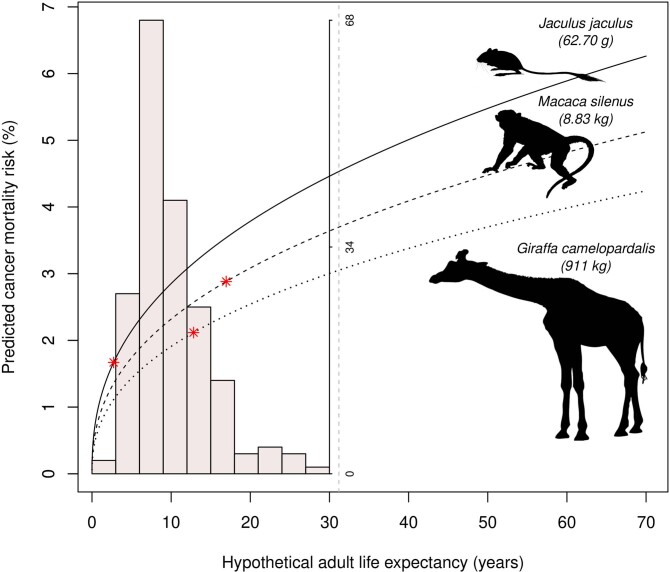
Predicted CMR at various hypothetical life expectancies for small, medium, and large-bodies species. CMR was predicted based on the logistic model presented in Extended Data Table 3a for a series of hypothetical adult life expectancies, ranging from one to 70 years (x-axis). Predictions were obtained for three body masses, corresponding to a small, medium and a large bodied mammal in our dataset. True life expectancies of the three species are marked with red stars. Distribution of life expectancies across the species set of the model is shown by a histogram. Vertical dashed line marks maximum life-expectancy in our dataset. *Jaculus jaculus* silhouette by Maija Karala. The other animal silhouettes used to visually represent mammalian orders were downloaded from PhyloPic (http://www.phylopic.org).

The probability of detecting cancer (CMR: *n* = 188; ICM: *n* = 141) (Extended Data Table 3 and Extended Data Figs. 4 and 5), as well as non-zero cancer mortality risk (CMR: *n* = 141; ICM: *n* = 128) (Extended Data Table 3 and Fig. 3), tended to decrease with larger body masses and to increase with longer life expectancies. These effects were not significant, and were consistent between the two cancer mortality metrics. These associations were also largely independent of each other, and were similar in single-predictor models (Supplementary Table 3). The effect of body mass on cancer risk was even slightly negative, but the models indicated only a 2.8–2.9% decrease in cancer risk for a doubling of the body weight (for example, CMR changes from 3.82% (1 kg) to 3.71% (2 kg), or from 2.86% (1,024 kg) to 2.78% (2,048 kg), respectively, predictions obtained from the model presented in Extended Data Table 3, with an arbitrary life expectancy of 27 years, Extended Data Fig. 6). Additionally, body mass accounted for only 0.78% of the cross-species variance in CMR (that is, partial coefficient of determination). Similarly, cancer mortality risk only minimally and nonsignificantly increased with higher life expectancy, indicating a 24.7–25.2% increase in cancer risk for a doubling of the adult life expectancy (for example, CMR changes from 0.89% (1 year) to 1.18% (2 years), or from 2.80% (16 years) to 3.72% (32 years), respectively, predictions obtained from the model presented in Extended Data Table 3 with an arbitrary body mass of 10 kg, Extended Data Fig. 6). Adult life expectancy accounted for only 2.94% of the variance observed in CMR (that is, partial coefficient of determination). Overall, these results provide the largest-scale and most robust support to the body size and life expectancy components of Peto’s paradox in mammals. They suggest that lifespan extension and larger body size jointly evolved with better anticancer mechanisms across mammals.

Since the first indication of species differences in cancer predisposition, an intense search has been conducted to identify mechanisms explaining cancer resistance in certain species, mostly rodents^35^, and very large animals^17^. Although these studies demonstrated key species-specific anticancer mechanisms^17,28,35^, a considerable gap remains in our knowledge on the taxonomic and phylogenetic diversity and distribution of tumour-suppressing mechanisms. Our results provide a solid foundation for future studies scrutinizing these questions, by providing information on the generality and frequency of oncogenic phenomena across the mammalian phylogeny. We also highlight the exceptional resources provided by zoos for studies of cancer in wildlife^35,36^.

Our study indicates that death due to oncogenic phenomena is frequent and taxonomically widespread in mammals. In some species more than 20–40% of the managed adult population die of cancer-related pathologies. This estimate is staggering, especially knowing that cancer incidences estimated here are conservative (Methods). This observation urges the extensive exploration of cancer in wildlife, especially in the context of recent environmental perturbations^38^, as serious threats to animal welfare^29^.

## Methods

Documenting cancer in wild animals is extremely challenging in most cases owing to the lack of information on the age of individuals, the difficulty retrieving the bodies for necropsy and the likelihood of cancer negatively influencing survival before cancer itself could be detected. Although data on cancer incidence from wild populations would be indispensable to describe natural incidences of malignancies, such data, especially with corresponding ages and demographic histories, are unfortunately still far from our reach. Therefore, to estimate cancer mortality risk, we used data provided by Species360 and the Zoological Information Management System (ZIMS, Data Use Approval Number 73836), an international non-profit organization that maintains a real-time and centralized database of animals under human care (regrouping information from over 1,200 zoos worldwide). Although we recognize that the interpretation of data gathered on zoo animals requires caution, owing to strong human control on the diet, health, mortality factors, environment or standard biological functions of the animals, zoos provide exceptionally high data resolution on the demography and cause of death for a wide range of species. Here we rely on the high probability of body retrieval of deceased zoo animals and the necropsy routinely performed on most of them (unless found in an advanced stage of decomposition), aiming to identify the most likely pathology causing the death of the animal. These examinations are likely to reveal most solid tumours, but (although possible) benign tumours, liquid tumours (for example, leukaemia) or early-stage cancers are unlikely to be recorded here, either owing to their diagnostic difficulty or their perceived limited role in contributing to the death of the animal.

Specifically, here we use the husbandry module of ZIMS, providing information on birth, death, sex and pre-defined categories of pathological findings, including neoplasia (by definition tumours that contributed to the death, albeit with no option to specify the cancer type or other details). No statistical methods were used to predetermine sample size, but to minimize bias caused by potential temporal heterogeneity in data management practices and necropsy record-keeping^15^, here we focused on individuals alive or born after 1 January 2010 (data extraction: 30 May 2020). This sample was then used to characterize species-specific life expectancies and cancer incidence, but only after the exclusion of data that did not fulfil a series of criteria, to ensure the highest and most homogeneous data quality possible. First, cancer is an age-related disease that rarely occurs in juveniles, and pediatric cancers are usually medically distinct from adults’ cancers. As such, infant mortality differences observed across species can significantly confound cancer incidence estimates. Therefore, we gathered sex-specific or species-specific (wherever the former was not available) ages at sexual maturity and we considered individuals for analyses only if they reached maturity before or during our sampling period. For individuals of unknown sex (about 12% of all individuals in the raw data extract), the maximum of ages at sexual maturity of males and females was used as an inclusion age threshold. Sex-specific age at sexual maturity for each species was obtained from Conde et al.^19^ or from published literature resources (see data sources in https://github.com/OrsolyaVincze/VinczeEtal2021Nature/blob/main/SupplementaryData.xls). Second, given that age is a key predictor of cancer emergence, we considered only individuals for which date of birth was recorded precisely or within a narrow (maximum 30 days) time interval. Third, we considered only species in which postmortem pathological records were available for at least 20 adult individuals, irrespective of the cause of death (for example, infection, accident, geriatric disease and so on). Nonetheless, models presented were performed with increased thresholds of 40, 60, 80 and 100 individuals to check for consistency in the results (Supplementary Table 2). Fourth, given that the process of domestication is widely regarded as a major contributing factor to inbreeding depression and higher incidence of cancer^39^, we excluded all species that were subject to domestication as well as their wild ancestors (taxa excluded owing to being subject to domestication are listed in Supplementary Table 1). Following these restrictions, data extraction on age and cause of death resulted in information for 110,148 (62,556 live and 47,592 dead) individuals (*n* = 191 species). For calculation of ICM, we included only species in which survival is correctly estimated until old ages (that is, data allowing the estimation of age-specific survival until the age at which only 10% of individuals are surviving, *n* = 172 species). While these restrictions removed multiple sources of bias in our cancer mortality risk estimates, we cannot exclude the possibility that some species (for example, more charismatic ones) are subject to more frequent or more detailed necropsies. Nonetheless, our statistical approach, especially the complete case analysis, is largely insensitive to such biases, as individuals not having available postmortem diagnostic records are considered censored (see below). Also, while the depth of necropsy might vary slightly among species, neoplasia that had a significant contribution to the death of the animals (the focus of our study) are generally detected even at gross necropsies. Additionally, large species are considered of key importance for zoos, also reflected by the fact that the proportion of dead individuals with postmortem pathological records is larger in larger species (Pearson’s correlation: *r* = 0.24, *t* = 3.35, df = 189, *P* = 0.001). Accordingly, if charisma had a role in cancer detection, we would expect a larger cancer risk in large mammals, opposite to the (nonsignificant) negative body mass effect in our models. Consequently, we believe that charisma is unlikely to represent a major source of bias in our analysis.

### Estimation of adult life expectancy

As we have no reason to believe that censored individuals would not have the same prospect of survival as those who continue to be followed, we estimate adult life expectancy from age-specific survival estimated using the Kaplan–Meier procedure (using the survfit function in the R package survival^40^). Individuals older than their age at sexual maturity on 1 January 2010 were left-truncated at their age at this date; individuals reaching sexual maturity after this date were left-truncated at their age at sexual maturity. Individuals still alive at the time of data extraction were considered right-censored (samples per species varied from 42 to 5,816 individuals), while known fate individuals were assigned as dead (*n* = 47,592), irrespective of whether their cause of death was specified or not.

### Estimation of ICM

ICM was calculated using a competing risk approach, based on the cumulative hazard of cancer-related deaths and survival probability of the species under human care. First, age-specific survival *S*_*x*_, at age *x*, was estimated from KM analysis as above. However, here we performed a complete-case analysis, using only 11,840 individuals for which the cause of death was specified together with right-censored survivors. Complete-case analysis assumes that missingness in the cause of failure is random, but we had no reason to believe that this was not the case in our dataset. Postmortem examinations are routinely carried out on most recovered bodies in zoos, and once examinations are performed the results are equally likely to be entered in the database irrespective of the pathologies identified. ICM estimates were thus based on *n* = 74,396 individuals, *n* = 179 species. Second, the cancer mortality hazard *h*^c^_*x*_ was estimated using a KMx1 analysis where only deaths by cancer were incorporated as a death event. ICM is then such that

$${\rm{I}}{\rm{C}}{\rm{M}}=\mathop{\sum }\limits_{x=\alpha }^{{\rm{\infty }}}{S}_{x}{h}_{x}^{{\rm{c}}}$$

where *α* is the age at sexual maturity. The only difference with classic estimation is that we extracted *h*^c^_*x*_ (and *S*_*x*_) for each time unit with discrete jumps (and falls) at event times at age *t* and with *h*^c^_*t≤x<t+*1_ = 0 (*S*_*x*_ constant) between these events; instead of estimating discrete hazard ${h}_{t}^{{\rm{c}}}={d}_{t}^{{\rm{c}}}/{n}_{t}$ on these time intervals (where *d*^c^_*t*_ is the number of deaths by cancer within the interval and *n*_*t*_ is the number of survivors at the beginning of the interval). We chose this method to reflect true variation in the data for the interspecific comparison where species differ greatly in the number of events and time interval between these (sometimes a third of the organism’s adult lifespan), a situation rarely met when comparing human groups.

### Covariates and statistical analyses

For each species, we obtained sex-specific adult body mass data from Species360’s ZIMS (see https://github.com/OrsolyaVincze/VinczeEtal2021Nature/blob/main/SupplementaryData.xls). Species-specific body mass was calculated as the average of all body mass measurements recorded in the ZIMS database in adults, while species-specific values were obtained by averaging the body masses of males and females. These were calculated only in species for which there were at least 100 adult body mass records; otherwise, body mass was taken from the literature and database review by Conde et al.^19^. We verified that there was a one-to-one correspondence in the body mass information for species with records in both datasets.

Diet information was obtained from a global diet dataset for terrestrial mammals^32^, providing information on diet composition at four hierarchical levels of food items (never consumed, occasionally consumed, secondary food item, primary food item). We collected information on animal content in diet, as well as subcategories of this diet class, namely invertebrate or vertebrate consumption, as well as specifically fish, reptile, bird and mammal consumption. Given that most food items had few species in the intermediate levels (occasional consumption and secondary food item), we re-categorized the diet variables at two levels: never/rarely consumed or representing the primary/secondary food item of the species. The effect of diet was tested in PGLS regressions using only species with non-zero cancer mortality risks. Models were run separately for each food item that were entered to a base model including body mass and adult life expectancy as covariates. Results are shown in Extended Data Table 2.

To account for statistical non-independence due to phylogenetic relationships, we obtained a sample of 1,000, equally plausible phylogenetic trees, from the posterior distribution published by Upham et al.^41^, covering 5,911 species. We then obtained a rooted consensus tree using the sumtrees Python library^42^. Two species recently raised to species level were manually added to the tree as sister taxa of the species it was recently separated from (that is, *Cervus canadensis* to *Cervus elaphus* and *Gazella marica* to *Gazella subgutturosa*). Phylogenetic signal of cancer risk was assessed using the function phylosig from the R package phytools^22^. Partial coefficients of determinations were calculated using the function R2.pred from the R package rr2 (ref. ^43^), based on models presented in Extended Data Table 3.

**Extended Data Fig. 7:**
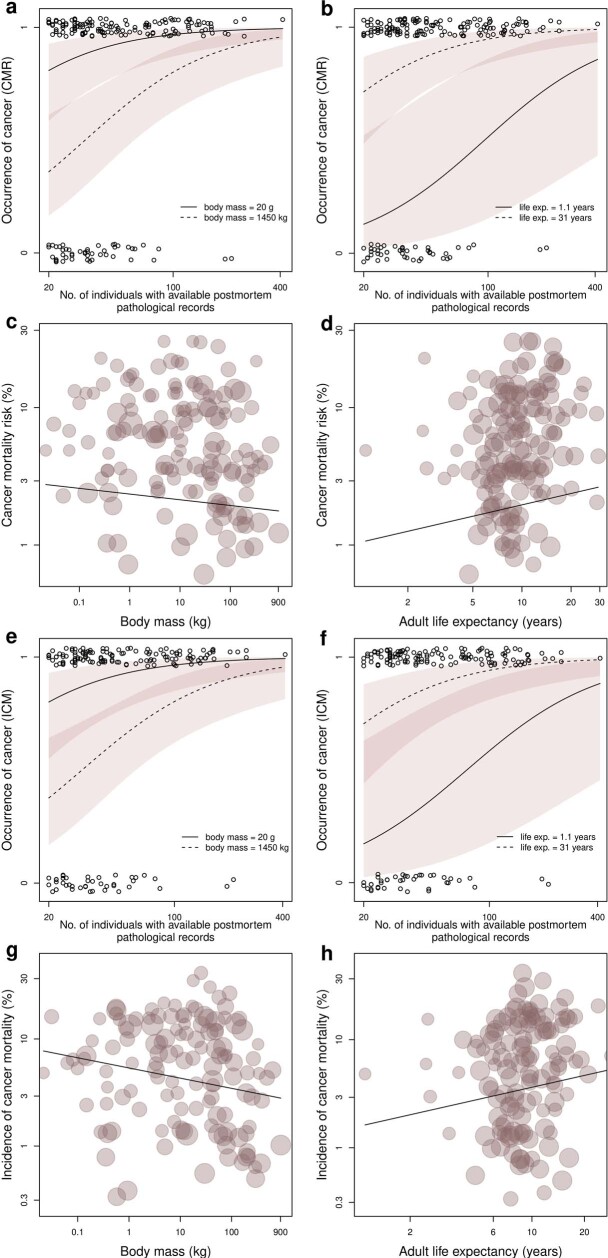
Association between cancer mortality risk and body mass as well as adult life expectancy across 191 mammal species. These analysis include the three species excluded in some models due to their high leverages. Plots show **a-d**, CMR or **e-h**, ICM. Occurrence of cancer in each species is plotted in function of the number of individuals with post-mortem pathological records. Predictions were obtained for two scenarios: for small or large **a,e**, body masses and low or high **b,f**, adult life expectancies respectively. Random noise was added to cancer occurrence to facilitate the visualization of overlapping points. Predictions and associated 95% confidence intervals were obtained from a binomial GLM without phylogenetic control. Non-zero cancer mortality risks were plotted against **c,g**, body mass or **d,h**, adult life expectancies. Slopes were obtained from the PGLS model presented in Supplementary Table 4. Points are proportional to the log number of individuals with known cause of death.

Models of cancer risk testing Peto’s paradox were performed using zero-inflated logistic models, which allow us to make inferences on the probability of detecting at least one cancer case in the species and, given that cancer was detected, inferences on the CMR or ICM. Therefore, the first part of this consisted of a phylogenetic binomial regression (using the function binaryPGLMM, in the R package ape^44^), where the dependent variable explained the presence of zeros and non-zeros in CMR or ICM. This model contained the log number of deceased individuals with available postmortem pathological records as an explanatory variable, due to the higher probability of detecting cancer with a higher number of dead individuals inspected. Additionally, the model contained body mass and adult life expectancy as covariates. The second part of the model consisted of a PGLS regression that investigated variance only in non-zero cancer risks. ICM and CMR were logit-transformed in all PGLS models as recommended when analysing proportions^45^. These models were weighted by log number of deceased individuals with available postmortem pathological records, as the precision of cancer mortality risk estimates is expected to increase with the number of dead individuals subject to postmortem examination, but it is not expected to explain bias in the estimation of the dependent variable in any particular direction (as in the case of the binomial models). These models also contained body mass and adult life expectancy as explanatory variables. Given the expected additive effect of body mass and longevity, the interaction between body mass and longevity metrics was also tested in all four models (binomial and logistic regressions for CMR and ICM), but these interactions did not increase model fit in any case and are therefore not presented. Both models were controlled for phylogenetic relatedness among species, where the scaling parameter of phylogenetic dependence (that is, s2/Pagel’s *λ* in PGLMMs and PGLSs respectively) was set to the most appropriate values assessed by likelihood ratio statics in each model separately. PGLS models in which Pagel’s *λ* converged to negative values were refitted with Pagel’s *λ* fixed at 0. Three species (*Lagurus lagurus*, *Cricetus cricetus* and *Dasyuroides byrnei*) had been removed from the latter models, due to their high leverage caused by their very low adult life expectancy compared to the rest of the species and therefore concerns of strong influence of these points over model fit. Nonetheless, all models were performed using the entire dataset, and the results were highly consistent with and without the exclusions (Supplementary Table 4 and Extended Data Fig. 7).

Order differences in cancer incidence were tested using standard linear regressions, built using only taxonomic orders in which at least two species had their cancer incidence estimated. The model contained CMR or ICM (non-transformed) as dependent variables and order as the sole explanatory factor. Pairwise order differences were assessed using the R package emmeans^46^. All analysis were performed in the R Statistical and Programming Environment, version 4.0.4 (ref. ^47^). Cancer mortality risks were transformed to percentages in figures and in the analysis performed on order differences (Extended Data Table 1), for easier interpretation. Models presented in Extended Data Tables 2 and 3 and Supplementary Tables 2–4 are based on probabilities.

### Reporting summary

Further information on research design is available in the Nature Research Reporting Summary linked to this paper.

## Online content

Any methods, additional references, Nature Research reporting summaries, source data, extended data, supplementary information, acknowledgements, peer review information; details of author contributions and competing interests; and statements of data and code availability are available at 10.1038/s41586-021-04224-5.

### Supplementary information

Supplementary Tables This file contains Supplementary Tables 1–4, which include a list of species excluded from the analyses due to being subject to domestication (Table 1), and sensitivity analyses performed to test the effect of within-species sample size (Table 2), test for collinearity issues (Table 3) and confirm the consistency of the results when high-leverage species are included in the analyses (Table 4).

Reporting Summary

## Figures and Tables

**Extended Data Table 1:**
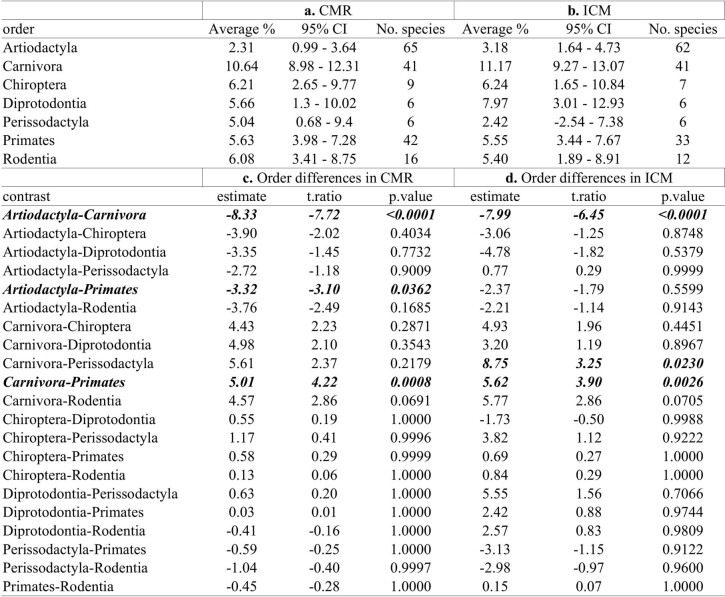
Order differences in cancer mortality risk

measured as **a**, CMR or **b**, ICM. Only orders represented by a minimum of two species were included in these analyses. Models were constructed using R function "lm" using cancer mortality risks as a dependent variables and order as a sole predictor. Values presented are estimated marginal means and their associated 95% confidence intervals. The number of species for which data was available in each order is also shown. Post-hoc test of order differences in **c**, CMR or **d**, ICM across mammalian orders are shown.

**Extended Data Table 2:**
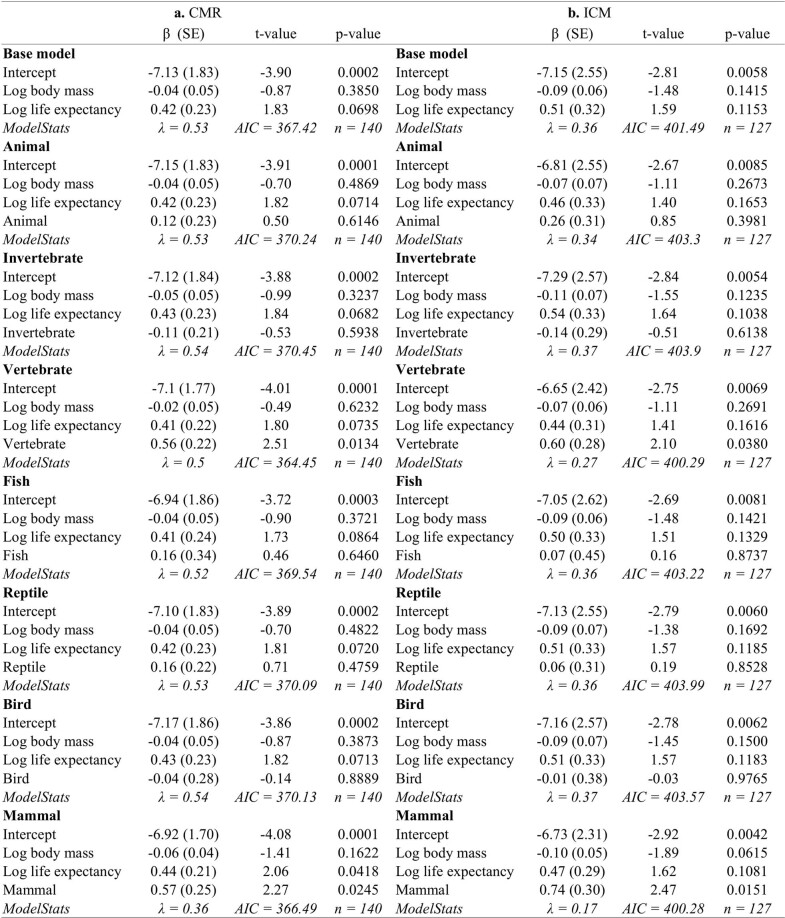
Association between cancer risk and diet animal content

Models explain variance in **a**, CMR or **b**, ICM in function of body mass, life expectancy and diet animal content. Diet animal content is characterized by variables on three taxonomic level: 1. animal content (including any vertebrate or invertebrate prey); 2. invertebrate or vertebrate prey; and 3. within vertebrates fish, reptiles, birds and mammals. Each diet item is coded as a two-level factor: rarely/never occurring in diet and representing the primary/secondary food item of the species. Each diet variable is added one by one to a base model containing the two significant predictors of cancer risk: body mass and life expectancy. Akaike’s Information Criteria (AIC) are directly comparable among models with the same independent variable. All models are PGLS regressions.

**Extended Data Table 3:**
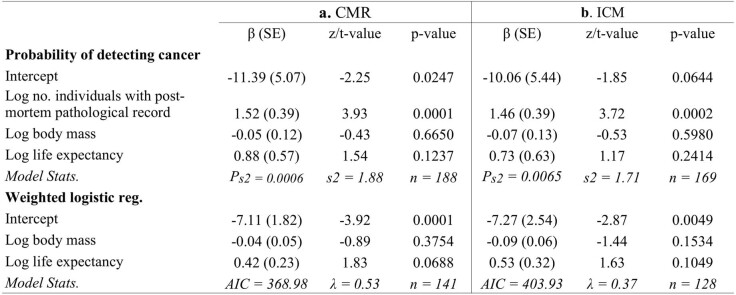
Results of phylogenetic models exploring variation in of cancer mortality risk

Results are presented for both **a**, CMR (%) and **b**, ICM (%). Binomial phylogenetic GLMMs are presented first and phylogenetic GLSs exploring variation in non-zero cancer mortality risks are presented second. For each model sample size (n) and phylogenetic inertia (s2/λ) are presented at the bottom of the results.

## Data Availability

Data used for the analysis presented in the paper are available at https://github.com/OrsolyaVincze/VinczeEtal2021Nature/blob/main/SupplementaryData.xls. Raw data used to estimate cancer risk (Species360 Data Use Approval Number 73836) cannot be publicly shared, as Species360 is the custodian (not the owner) of their members’ data. Raw data are accessible through Research Request applications (form available at https://docs.google.com/forms/d/1znoy62VEkDlhAp_0RfEvF7Zsx03g4W5AlppJHqo3_WQ/viewform?edit_requested=true&pli=1). Research Requests are reviewed by both the Species360 Research Committee and their Board of Trustees every four months. The Board of Trustees makes the final decision on data sharing, based on recommendations by the Research Committee. Once Species360 grants access to data, they are intended only for and restricted to use in the project they were approved for and for a single publication. The researcher cannot use them for other projects, publications and/or purposes, nor can the researcher share the data with third parties. For any other inquires, all of the details for the submission of research requests to Species360 can be found at https://conservation.species360.org/wp-content/uploads/2020/08/Species360-Sharing-Data-v3-3_komprimeret.pdf. Any email communications should be directed to support@species360.org.

## Code availability

Data and R code needed to reproduce the analysis are publicly available at https://github.com/OrsolyaVincze/VinczeEtal2021Nature.
