# Supplementary material for: Cancer risk across mammals
Source: Nature. 2021 Dec 22;601(7892):263–7. doi: 10.1038/s41586-021-04224-5 (PMC8755536; doi:10.1038/s41586-021-04224-5)
Supplement: Supplementary file 1 — This file contains Supplementary Tables 1–4, which include a list of species excluded from the analyses due to being subject to domestication (Table 1), and sensitivity analyses performed to test the effect of within-species sample size (Table 2), test for collinearity issues (Table 3) and confirm the consistency of the results when high-leverage species are included in the analyses (Table 4). [file 41586_2021_4224_MOESM1_ESM.pdf]

nature portfolio

https://doi.org/10.1038/s41586-021-04224-5

Supplementary information

Cancer risk across mammals

In the format provided by the authors and unedited

Supplementary Table 1 | List of species excluded from the analyses due to being subject to domestication. Domestication is likely to affect cancer risk, body mass or life expectancy.

Species	order
Atelerix_albiventris	Erinaceomorpha
Bos_gaurus	Artiodactyla
Bos_grunniens	Artiodactyla
Bos_javanicus	Artiodactyla
Bos_taurus	Artiodactyla
Camelus_bactrianus	Artiodactyla
Canis_lupus	Carnivora
Capra_hircus	Artiodactyla
Cavia_porcellus	Rodentia
Chinchilla_lanigera	Rodentia
Equus_africanus	Perissodactyla
Equus_asinus	Perissodactyla
Equus_caballus	Perissodactyla
Felis_catus	Carnivora
Lama_glama	Artiodactyla
Lama_guanicoe	Artiodactyla
Mephitis_mephitis	Carnivora
Mustela_putorius	Carnivora
Oryctolagus_cuniculus	Lagomorpha
Ovis_aries	Artiodactyla
Rattus_norvegicus	Rodentia
Sus_scrofa	Artiodactyla
Vicugna_pacos	Artiodactyla
Vicugna_vicugna	Artiodactyla

Supplementary Table 2. - Sensitivity analysis performed to test the effect of within-species sample size. Models were performed to explore the consistency of results of weighted logistic regressions of non-zero cancer risks when using a different minimum threshold of number of individuals with available postmortem pathological records. Models were run with a minimum of 20, 40, 60, 80 and 100 individuals with available postmortem pathological records per species (N). Slopes, standard errors and p values are presented for all models. Please note the considerable decrease in the number of species involved in the analyses (n) with increased within-species threshold sample sizes.

	**Number of individuals with available post-mortem pathological record**	**N ≥ 20**	**N ≥ 40**	**N ≥ 60**	**N ≥ 80**	**N ≥ 100**
		**β ± SE [p-value]**	**β ± SE [p-value]**	**β ± SE [p-value]**	**β ± SE [p-value]**	**β ± SE [p-value]**
**CMR**	**Weighted logistic reg.**					
	Intercept	-7.11 ± 1.82 [0.0001]	-4.53 ± 2.8 [0.1106]	-5.4 ± 3.76 [0.1567]	-3.36 ± 4.49 [0.4589]	-2.55 ± 5.01 [0.6148]
	Log body mass	-0.04 ± 0.05 [0.3754]	0.04 ± 0.07 [0.5242]	0.04 ± 0.09 [0.6367]	0.06 ± 0.10 [0.5793]	0.02 ± 0.11 [0.8559]
	Log life expectancy	0.42 ± 0.23 [0.0688]	0.12 ± 0.36 [0.7491]	0.25 ± 0.48 [0.6037]	-0.01 ± 0.57 [0.9916]	-0.09 ± 0.64 [0.8924]
	Number of species	n = 141	n = 81	n = 56	n = 42	n = 32
**ICM**	**Weighted logistic reg.**					
	Intercept	-7.27 ± 2.54 [0.0049]	-4.8 ± 4.03 [0.2378]	-4.04 ± 5.17 [0.4388]	-3.73 ± 6.35 [0.5611]	-3.20 ± 6.61 [0.6321]
	Log body mass	-0.09 ± 0.06 [0.1534]	-0.05 ± 0.10 [0.6025]	-0.07 ± 0.12 [0.5654]	-0.07 ± 0.13 [0.6208]	-0.15 ± 0.15 [0.3238]
	Log life expectancy	0.53 ± 0.32 [0.1049]	0.22 ± 0.52 [0.677]	0.13 ± 0.66 [0.8395]	0.10 ± 0.80 [0.9002]	0.08 ± 0.84 [0.9291]
	Number of species	n = 128	n = 77	n = 55	n = 41	n = 32

Supplementary Table 3. - Sensitivity analysis performed to test for collinearity issues. In order to demonstrate the lack of collinearity in models presented in Extended Data Table 3, here we present the results of models explaining variation in **a-b**, CMR and **c-d**, ICM, where body mass (**a**,**c**) and life expectancy (**b**,**d**) are entered in separate models.

	**a.** Effect of body mass on CMR		
	β (SE)	z/t-value	p-value
**Probability of detecting cancer**			
Intercept	-4.09 (1.65)	-2.48	0.0131
Log no. individuals with post-mortem pathological record	0.06 (0.10)	0.64	0.5201
Log body mass	1.41 (0.38)	3.77	0.0002
ModelStats	*Ps2 < 0.0001*	*s2 = 2.31*	*n = 188*
**Weighted logistic reg.**			
Intercept	-4.00 (0.47)	-8.55	0.0000
Log body mass	0.01 (0.04)	0.15	0.8784
ModelStats	*AIC = 369.14*	*λ = 0.57*	*n = 141*

	**b**. Effect of life expectancy on CMR		
	β (SE)	z/t-value	p-value
**Probability of detecting cancer**			
Intercept	-10.21 (4.24)	-2.41	0.0161
Log no. individuals with post-mortem pathological record	1.49 (0.38)	3.93	0.0001
Log life expectancy	0.74 (0.47)	1.59	0.1130
ModelStats	*Ps2 = 0.0002*	*s2 = 1.9*	*n = 188*
**Weighted logistic reg.**			
Intercept	-6.45 (1.62)	-3.97	0.0001
Log life expectancy	0.32 (0.2)	1.6	0.1115
ModelStats	*AIC = 363.5*	*λ = 0.56*	*n = 141*

	**c.** Effect of body mass on ICM		
	β (SE)	z/t-value	p-value
**Probability of detecting cancer**			
Intercept	-4.08 (1.67)	-2.45	0.0144
Log no. individuals with post-mortem pathological record	1.39 (0.38)	3.62	0.0003
Log body mass	0.03 (0.1)	0.26	0.7923
ModelStats	*Ps2 = 0.0004*	*s2 = 2.12*	*n = 169*
**Weighted logistic reg.**			
Intercept	-3.22 (0.50)	-6.41	<0.0001
Log Body mass	-0.04 (0.06)	-0.67	0.5063
ModelStats	*AIC = 404.15*	*λ = 0.39*	*n = 128*

	**d**. Effect of life expectancy on ICM		
	β (SE)	z/t-value	p-value
**Probability of detecting cancer**			
Intercept	-8.45 (4.50)	-1.88	0.0603
Log no. individuals with post-mortem pathological record	1.42 (0.39)	3.70	0.0002
Log life expectancy	0.54 (0.51)	1.06	0.2875
ModelStats	*Ps2 = 0.0020*	*s2 = 1.75*	*n = 169*
**Weighted logistic reg.**			
Intercept	-5.62 (2.27)	-2.47	0.0148
Log life expectancy	0.29 (0.28)	1.03	0.3029
ModelStats	*AIC = 400.26*	*λ = 0.4*	*n = 128*

Supplementary Table 4. - Sensitivity analysis performed to confirm the consistency of the results when high leverage species are included in the analyses. In order to demonstarte the consistency of the result while including the three species excluded from the analyses presented in the main text due to their high leverages (i.e. *Lagurus lagurus*, *Cricetus cricetus*, and *Dasyuroides byrnei*), here we present models exploring the association between **a**, CMR and **b**, ICM and body mass as well as life expectancy on the entire species pool.

	**a. CMR**			**b. ICM**		
	β (SE)	z/t-value	p-value	β (SE)	z/t-value	p-value
**Probability of detecting cancer**						
Intercept	-8.07 (4.71)	-1.71	0.0870	-6.33 (5.01)	-1.26	0.2070
Log no. individuals with post-mortem pathological record	1.44 (0.38)	3.79	0.0002	1.37 (0.38)	3.57	0.0004
Log body mass	-0.03 (0.12)	-0.27	0.7892	-0.05 (0.13)	-0.37	0.7126
Log life expectancy	0.53 (0.54)	0.97	0.3302	0.33 (0.59)	0.55	0.5791
Model Stats.	*Ps2<0.0001*	*s2 = 2.22*	*n = 191*	*Ps2 = 0.0010*	*s2 = 2.04*	*n = 172*
**Weighted logistic reg.**						
Intercept	-6.06 (1.84)	-3.30	0.0012	-5.94 (2.41)	-2.47	0.0150
Log body mass	-0.04 (0.05)	-0.84	0.4023	-0.10 (0.06)	-1.53	0.1282
Log life expectancy	0.29 (0.23)	1.23	0.2220	0.38 (0.31)	1.23	0.2210
Model Stats.	*AIC = 387.92*	*λ = 0.61*	*n = 144*	*AIC = 417.41*	*λ = 0.39*	*n = 131*
