# Supplementary material for: Cancer risk across mammals
Source: Nature. 2021 Dec 22;601(7892):263–7. doi: 10.1038/s41586-021-04224-5 (PMC8755536; doi:10.1038/s41586-021-04224-5)
Supplement: Supplementary file 2 — Reporting Summary [file 41586_2021_4224_MOESM2_ESM.pdf]

nature portfolio

Corresponding author(s): Orsolya Vincze

Last updated by author(s): Sep 24, 2021

# Reporting Summary

Nature Portfolio wishes to improve the reproducibility of the work that we publish. This form provides structure for consistency and transparency in reporting. For further information on Nature Portfolio policies, see our Editorial Policies and the Editorial Policy Checklist.

## Statistics

For all statistical analyses, confirm that the following items are present in the figure legend, table legend, main text, or Methods section.

| n/a | Confirmed | |
|---|---|---|
| ☐ | ☒ | The exact sample size (*n*) for each experimental group/condition, given as a discrete number and unit of measurement |
| ☒ | ☐ | A statement on whether measurements were taken from distinct samples or whether the same sample was measured repeatedly |
| ☐ | ☒ | The statistical test(s) used AND whether they are one- or two-sided<br>*Only common tests should be described solely by name; describe more complex techniques in the Methods section.* |
| ☐ | ☒ | A description of all covariates tested |
| ☒ | ☐ | A description of any assumptions or corrections, such as tests of normality and adjustment for multiple comparisons |
| ☐ | ☒ | A full description of the statistical parameters including central tendency (e.g. means) or other basic estimates (e.g. regression coefficient) AND variation (e.g. standard deviation) or associated estimates of uncertainty (e.g. confidence intervals) |
| ☐ | ☒ | For null hypothesis testing, the test statistic (e.g. *F*, *t*, *r*) with confidence intervals, effect sizes, degrees of freedom and *P* value noted<br>*Give P values as exact values whenever suitable.* |
| ☒ | ☐ | For Bayesian analysis, information on the choice of priors and Markov chain Monte Carlo settings |
| ☒ | ☐ | For hierarchical and complex designs, identification of the appropriate level for tests and full reporting of outcomes |
| ☐ | ☒ | Estimates of effect sizes (e.g. Cohen's *d*, Pearson's *r*), indicating how they were calculated |

*Our web collection on statistics for biologists contains articles on many of the points above.*

## Software and code

Policy information about availability of computer code

| | |
|---|---|
| Data collection | No software or code was used to collect the data. All raw data was obtained from Species360 (a non-profit organization centralizing zoo data), through a research request. Raw data can be obtained directly from the organization (see https://www.species360.org/serving-conservation/research-data-request/) |
| Data analysis | All data analysis and graphical presentation was performed using R Statistical and Programming Environment, version 4.0.4<br>We estimated species-specific adult life expectancies from age-specific survival, estimated using a Kaplan-Meier procedure (using the “survfit” function in R package “survival” v. 3.2-11.<br>Models of cancer risk were performed using zero-inflated logistic models. The first part of this model consisted of a phylogenetic binomial regression (using the function binaryPGLMM, in R package "ape" v. 5.5), the second part of the model consisted of a phylogenetic generalised least squares regression (PGLS, built using the function gls in R package "nlme" v. 3.1-153).<br>Phylogenetic signal of cancer risk was assessed using the function ‘phylosig’ from R package ‘phytools’ v. 0.7-80.<br>All packages and functions are described and fully referenced in the methods.<br>Consensus phylogenetic tree was obtained using the ‘sumtrees’ library (v. 4.4.0) in python (v. 2.7.16).<br><br>All codes used to generate the presented results are available on https://github.com/OrsolyaVincze/VinczeEtal2021Nature. |

For manuscripts utilizing custom algorithms or software that are central to the research but not yet described in published literature, software must be made available to editors and reviewers. We strongly encourage code deposition in a community repository (e.g. GitHub). See the Nature Portfolio guidelines for submitting code & software for further information.

## Data

Policy information about availability of data

All manuscripts must include a data availability statement. This statement should provide the following information, where applicable:

- Accession codes, unique identifiers, or web links for publicly available datasets
- A description of any restrictions on data availability
- For clinical datasets or third party data, please ensure that the statement adheres to our policy

All data required for the replication of the analysis are results are given as Source Data. Raw data are in the possession of Species360 and are available through research request from the organization (https://www.species360.org/serving-conservation/research-data-request/).

# Field-specific reporting

Please select the one below that is the best fit for your research. If you are not sure, read the appropriate sections before making your selection.

☒ Life sciences ☐ Behavioural & social sciences ☐ Ecological, evolutionary & environmental sciences

For a reference copy of the document with all sections, see nature.com/documents/nr-reporting-summary-flat.pdf

# Life sciences study design

All studies must disclose on these points even when the disclosure is negative.

| | |
|---|---|
| Sample size | We worked with the widest database and largest sample size available at Species360. Species were considered for statistical analysis if minimum 20 individuals had postmortem pathological records. Models were however run by increasing this sample size to 40, 60, 80 and 100 to confirm the robustness of the results. Results of this sensitivity analysis is given in Supplementary Table 2. |
| Data exclusions | We excluded all species that were subject to domestication, because the process of domestication is widely regarded as a major contributing factor to inbreeding depression and higher incidence of cancer. We list in the species excluded due to this reason in Supplementary Table 1 and provide references in the manuscript summarizing the potential biases caused by domestication.<br>Due to their high leverages (unusually low life expectancies) three species (Lagurus lagurus, Cricetus cricetus, and Dasyuroides byrnei) were eliminated from the main analysis presented in the manuscript. The thee species are listed in the Methods section, and the reason for their exclusion is explained. All analysis were run however also including these three species to ensure that the results are robust and not affected by the above exclusion. Results of the latter sensitivity analysis are presented in Supplementary Table 4 and Extended Data Figure 7. |
| Replication | To ensure the replicability of the results, we estimated two different metrics of cancer mortality risk, both estimating the proportion of individuals dying of cancer. All models are presented using both metrics as dependent variables.<br>Moreover, to ensure the replicability of the results we performed sensitivity analysis using different minimum threshold of number of individuals with available postmortem pathological records. Results of these analyses are presented in Supplementary Table 2.<br>Where collinearity was plausible, models were re-run with single predictors to ensure that effects are consistent, as shown in Supplementary Table 3. |
| Randomization | Initial species pool used here, represented the widest available husbandry database of Species360 at the time of data extraction. Exclusion included species subject to domestication (as shown in Supplementary Table 1) and three species whose leverage was concerning (all analysis were repeated including species, as presented in Supplementary Table 4).<br>Diet categories were pre-defined by an independent published work (Kissling et al. 2014 Ecol.Evol.) and were adopted without modifications. |
| Blinding | Diet categories were pre-defined by an independent published work (Kissling et al. 2014 Ecol.Evol.) and were adopted without modifications.<br>No other group allocation was necessary. |

# Reporting for specific materials, systems and methods

We require information from authors about some types of materials, experimental systems and methods used in many studies. Here, indicate whether each material, system or method listed is relevant to your study. If you are not sure if a list item applies to your research, read the appropriate section before selecting a response.

## Materials & experimental systems

| n/a | Involved in the study |
|---|---|
| ☒ | ☐ Antibodies |
| ☒ | ☐ Eukaryotic cell lines |
| ☒ | ☐ Palaeontology and archaeology |
| ☒ | ☐ Animals and other organisms |
| ☒ | ☐ Human research participants |
| ☒ | ☐ Clinical data |
| ☒ | ☐ Dual use research of concern |

## Methods

| n/a | Involved in the study |
|---|---|
| ☒ | ☐ ChIP-seq |
| ☒ | ☐ Flow cytometry |
| ☒ | ☐ MRI-based neuroimaging |
